# Merging *Fargesia dracocephala* into *Fargesia decurvata* (Bambusoideae, Poaceae): Implications from Morphological and ITS Sequence Analyses

**DOI:** 10.1371/journal.pone.0101362

**Published:** 2014-07-02

**Authors:** Yu-Qu Zhang, Xu-Mei Wang, A-Li Wu, Yi Ren

**Affiliations:** 1 College of Life Sciences, Shaanxi Normal University, Xi’an, China; 2 School of Pharmacy, Xi’an Jiaotong University, Xi’an, China; 3 Shaanxi Changqing National Nature Reserve, Huayang, China; Central China Normal University, China

## Abstract

**Aims:**

*Fargesia decurvata* is closely allied with *F. dracocephala* and differs in 5 major characters (i.e. the culm sheath blade base shape, the width of the culm sheath blade base, the auricle shape, and the lower surface of leaf blade) in *Fargesia*. It is difficult to distinguish these two species because of existing of transitional statements of characters. The aims of this paper are to (i) investigate whether the variation of the characters is continuous or not; (ii) reveal whether the publishment of *F*. *dracocephala* was the result of discontinuous sampling of *F*. *decurvata* or not.

**Methods:**

Ten populations of *F*. *decurvata* and *F*. *dracocephala* were investigated in their entire distribution (including type localities). The statements of 5 major characters were measured from 693 annual and 693 perennial culms of 231 individuals in 10 populations, and analyzed at population, individual and culm levels. UPGMA cluster analysis was carried out based on 29 characters from 10 populations of *F*. *decurvata* and *F*. *dracocephala* and 2 populations of *F*. *qinlingensis* as outgroup. The ITS sequences were also sequenced and analyzed.

**Important Findings:**

Five major characters exhibited great variation not only at population level, but at individual level within a population, even the culm level within an individual and in different parts of the same culm. Cluster analyses showed that 10 populations of *F*. *decurvata* and *F*. *dracocephala* were not divided into two species, but they were well separated with outgroup. There was no difference in floral organ between *F*. *decurvata* and *F*. *dracocephala*. MP and NJ trees based on ITS sequences showed the same results with the cluster analysis on morphological characters. All the facts indicated that the publishment of *F*. *dracocephala* was the result of discontinuous sampling of *F*. *decurvata*, and *F*. *dracocephala* should be treated as the synonym of *F*. *decurvata*.

## Introduction

The correct definition of species is the most basic work for further researches. The type and other voucher specimens play an important role in the process of defining a species. However, because the herbarium specimens are often collected from the limited location(s) and/or limited individual(s) or sometimes just from parts of an individual, the continuity of variation of character(s) within a species would be artificially isolated. The artificial isolation of character(s) might lead to the result that a virtual species will be defined as different ones [1]–[4].

The problems are particularly prominent in the definition of most of bamboo species. Because the bamboos typically have to go through over decades or even more than a century of vegetative growth before flowering, and generally died off within a few years after flowering, thus the chance to get the flowers and caryopses is very little when the specimens were collected, the defining and identifying of the bamboo species have been mainly depended on vegetative characters [5], [6]. Each of the bamboo is distributed in a wide range and grows in diverse habitat, but the specimens can be collected in limited locations and habitat, many vegetative variations might be ignored due to the limited sampling. In additional, only a small part of the culms, branches or rhizomes can be collected as the specimens. As a result, the specimen-based description and definition of bamboo species would inevitably cause the continuous variations of the characters to be fragmented.

**Figure 1:**
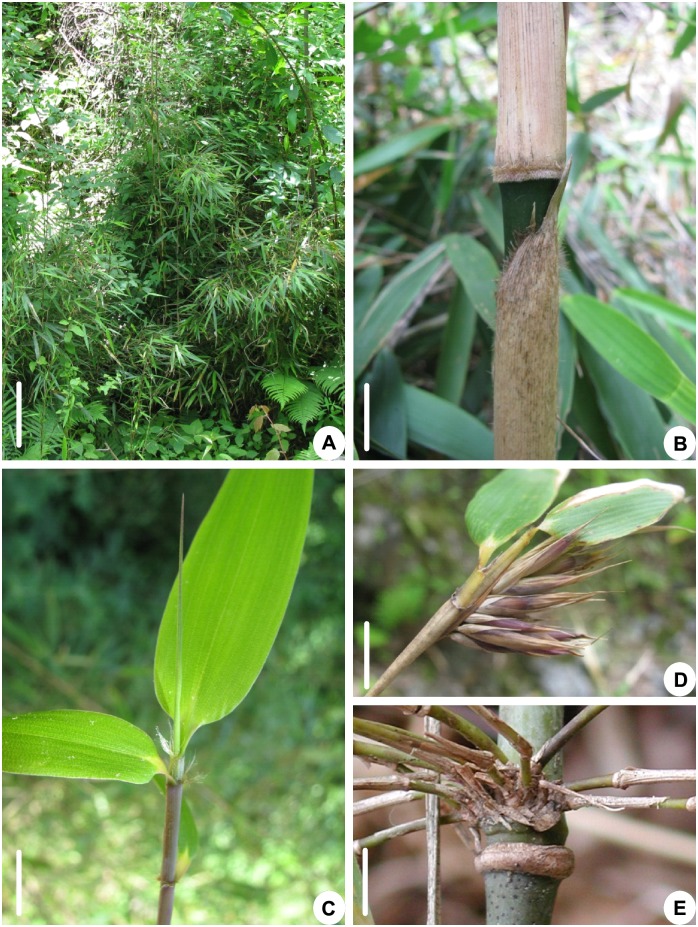
Morphology of *Fargesia decurvata*. A. Bamboo clump. Bar = 30 cm. B. Middle parts of a bamboo shoot. Bar = 1 cm. C. Branch and leaves. Bar = 0.8 cm. D. Yong infructescence. Bar = 1.5 cm. E. Close up of a node. Bar = 1 cm.

*Fargesia* is a large genus in Bambusoideae (Poaceae) with about 90 species distributed mainly in the alpine areas at altitude of 1400–3800 m in south-west China, Vietnam and adjacent Himalayas [7], [8]. There are 78 species in China and 61 of them were nominated and published without the description of reproductive organs and many of them are narrow distribution species [6]–[8]. In the field survey of *Fargesia* species as the main food of giant panda, we found that some bamboo samples from this genus could not be accurately identified because of the existing of transitional or intermediate characters between or among species. Among these species, *F. decurvata* Lu (Fig. 1) and *F. dracocephala* Yi are a pair of species which have wider distribution and are most difficult to identify. Therefore, we considered that these two species might be the good materials to study the definition of bamboo species.

*Fargesia decurvata* was described by Lu [9] based on specimen collected from Hejiaping, Changyang County, Hubei Province (Lu Jionglin 78120, Type in Herb. Honan Col. Agr. and PE). He considered that this species differs from other *Fargesia* species in following aspects: the base of the sheath blade is as wide as the sheath tongue or nearly so, the culm sheath tongue is promontory as a bow and strongly decurrently on both sides, the culm sheath has dense gray small bristles on the back and dense coarse wools at the base. There is no description of the reproductive organs. Thereafter Yi published *F. aurita*, which is based on specimens collected from Fengdu County, Sichuan Province (Yi Tongpei 75410, Type in Herb. Forestry School of Sichuan Province) [10], but the name was treated as a synonym of *F*. *decurvata* by Yi himself [7].

*Fargesia dracocephala* was described by Yi based on specimens collected from Guangwu Mountain, Nanjiang County, Sichuan Province (Yi Tongpei 75540, Type in Herb. Forestry School of Sichuan Province), Qianjiaping Forest Farm, Pingli County, Shaanxi Province and Shennongjia, Hubei Province [11]. He considered that this species differs from *F*. *robusta* in following aspects: the internodes are nearly solid and the hollows are 1–2.7 mm in diameter, the culm sheath is nearly hairless or with sallow thin bristles on the back, the leaf sheath is elliptic and with setae at apex, the leaf blade has 3–4 pairs of secondary veins. He described the morphology of flowers later [12].

*Fargesia decurvata* and *F. dracocephala* were considered as closely allied species both in *Flora Reipublicae Popularis Sinicae* (*FRPS*) [7] and Flora of China [8] and belong to *Fargesia* Sect. *Fargesia* Ser. *Yunnanenses*. In the field investigation on the giant panda’s food, we found that it is difficult to identify *F. decurvata* and *F. dracocephala* by the key characters because there are many transitional forms. When the previous references on these species were checked, we found that the description and illustration of two species were confused, especially in aspects of the shape of the leaf auricle and the position of the oral setae on the auricle. The only description on the auricle was “auriculae parvae” (auricle small) when Lu described *F*. *decurvata* for the first time [9]. Based on the observation on the type (Lu Jionglin 78120, PE), the auricles are mostly short strike-like with oral serae at margins or at margins and apex. When Yi (1985b) published *F*. *aurita* [10], which was treated as the synonym of *F*. *decurvata* [7], the auricles were described as “auriculis foliorum ellipticis” (leaf auricle elliptical), and there was no description on the seta position. However, figure in *FRPS* showed clearly that the setae are at the apex of the auricle. Thus, the shape of the leaf auricle should be short strike-like or elliptical with serae at the margins and/or apex. Later, the auricles of the species were described as “nearly circular” in *FRPS* [7] and Li *et al*. followed this description in Flora of China [8] and the setae were described as at the margins of the auricles [7], but the auricles seems not nearly circular but nearly short rectangle with setae at apex in Pl. 132, f. 17 in *FRPS* [7]. When *F*. *dracocephala* was described for the first time [11], the auricles were described as “auriculis foliorum ellipticis” (leaf auricle elliptical) and no description of the seta position, but it seems that the auricles are not elliptical but short banding with setae at apex from the illustration Fig. 4–6 in the paper of Yi [11].

In *FRPS*, Yi [7] described the auricles of the species as long elliptical but the shape of the auricles in the illustration Pl. 132, f.6 is the same with that in the paper of Yi [11], and this description was followed in Flora of China [8]. It seems that the variation of characters is various and might be continuous between two species. So we suspected that *F*. *decurvata* and *F*. *dracocephala* might be the representatives of two forms of a series morphological variation of vegetative organs. The publishing of the two species might be due to the discontinuous sampling of the same species.

In order to verify this question, the samples of *F. decurvata* and *F. dracocephala* from ten populations, which basically covered the total distribution of two species, were collected. The key characters plus 25 other characters of the two species were measured and analyzed to determine whether the variations of these characters are continuous or not. Furthermore, in order to confirm and enhance the variation pattern of vegetative morphologic characters, the sequences of the internal transcribed spacer (ITS) between the ribosomal RNA genes were used in the present studies because ITS has been one of the most frequent examples of DNA segments in plant systematic analysis. Although use of this multicopy region has several pitfalls, which have been summarized by [13], an indisputable practical advantage is the current abundance of information concerning ITS sequences in many taxa [14]–[20], enabling comparisons among different studies and numerous taxa. Here we attempted to integrate both morphological and molecular (ITS) data to assess the variation pattern of these characters between *F. decurvata* and *F. dracocephala*.

## Materials and Methods

### Ethics statement

According to regulations of the People’s Republic of China on the protection of wild plants, permits are required only for the species included in the list of state-protected plant species at the time of collection. Neither *Fargesia decurvata* and *F*. *dracocephala* nor *F*. *qinlingensis* is on the list of state-protected plant species (Yu YF, A milestone of wild plants protection in China - the first list of wild plants protected by the nation, Plants 1999 (5): 3–11; Regulations of the People’s Republic of China on the protection of wild plants, http://www.people.com.cn/item/faguiku/zrzyf/U1020.html). Thus, no specific permits were required for the described field studies. During the sample collection, only a part of culms and leaf blade were collected to avoid causing any harm to the plants and their habitats.

### Materials

The samples from ten populations of *F. decurvata* and *F. dracocephala*, including the populations from the type localities of two species, and two populations of *F*. *qinlingensis* Yi et J. X. Shao as the outgroup were collected from July to August in 2009 and 2010 (Table 1). Fifteen to twenty clumps (individuals) were randomly collected from each population, and the neighboring individuals were at least 5 m apart, so as to avoid resampling from the same individual. From each individual, 3–5 fresh leaves were sampled and immediately placed in silica gel, and stored at room temperature for DNA extraction. We collected seedlings of two to four years in Pop 3 and Pop 7 and floral organs in Pop 3, Pop 7 and Pop 9 among which Pop 3 can be identified as *F. decurvata* according to vegetative characteristics. The voucher specimens were deposited in the Herbarium of Shaanxi Normal University (SANU).

**Table 1:** Population information and ITS GenBank accession numbers.

Population No.	Species	Number of individual	Voucher	Location	Latitude (E)	Longitude (N)	Elevation (m)	ITS GenBank Acc. No.
Pop 1*	*F. decurvata*	15	ZYQ F634	Hejiaping, Changyang,Hubei	30°32′47.6″	110°32′48.4″	1535	JX841183
Pop 2	Approximate *F. dracocephala*	20	ZYQ A701	Niubeiliang, Zhashui,Shaanxi	31°24′58.8″	102°53′44.1″	1700	JX841184
Pop 3	Approximate *F. decurvata*	20	ZYQ A127	Longtanzi, Foping, Shaanxi	33°33′52.2″	107°54′20.2″	1530	JX841185
Pop 4	Approximate *F. dracocephala*	20	ZYQ A120	Changqing, Yangxian, Shaanxi	33°15′42.6″	107°33′29.9″	1580	JX841186
Pop 5	Approximate *F. dracocephala*	16	ZYQ A125	Huangbaiyuan, Taibai, Shaanxi	33°51′59.0″	107°33′00.2″	1823	JX841187
Pop 6	Approximate *F. dracocephala*	20	ZYQ A142	Zibaishan, Liuba, Shaanxi	33°40′34.2″	106°45′58.0″	2067	JX841188
Pop 7	Approximate *F. decurvata*	20	ZYQ A135	Shibandian, Liuba, Shaanxi	33°40′26.3″	106°45′31.3″	1890	JX841189
Pop 8*	*F. dracocephala*	20	ZYQ A134	Guangwushan, Nanjiang, Sichuan	32°39′39.7″	106°57′45.7″	1426	JX841190
Pop 9	Approximate *F. dracocephala*	20	ZYQ A702	Langhe, Zhenping, Shaanxi	32°01′19.3″	109°21′26.2″	1200	JX841191
Pop 10	Approximate *F. dracocephala*	20	ZYQ A627	Shennongjia, Fangxian, Hubei	31°28′16.5″	110°23′23.8″	1211	JX841192
Pop 11*	*F. qinlingensis*	20	ZYQ A071	Longtanzi, Foping, Shaanxi	32°40′48.6″	106°46′04.2″	1142	JX841193
Pop 12	*F. qinlingensis*	20	ZYQ A001	Taibaimiao, Ningshan, Shaanxi	33°25′07.5″	108°31′31.6	2030	JX841194

*: The population from type locality.

### Measurement and analysis of morphological characters

Based on *FRPS* [7] and Flora of China [8], five major characters, i.e. the base of the culm sheath blade is decurrent or not, the base of culm sheath blade is narrower than or as wide as the apex of the culm sheath, the auricle is nearly circular or oblong with setae at the apex or the margin, and the lower surface of leaf blade is setose or nearly glabrous, are used to identify *F. decurvata* and *F. dracocephala* (Table 2 and Table S1). Meanwhile, 24 expanded characters were also selected and measured (Table 3 and Table S1).

**Table 2:** The differences of the five major characters between *F. decurvata* and *F. dracocephala.*

Species/characters	Blade base of culm sheath	Width of bladebase of culmsheath	Leaf auricleshape	Position of oral setae on leaf auricle	Lower surface of leaf blade
*F. decurvata*	decurrent	as wide asculm sheath apex	nearly circular	at margin	pubescent
*F. dracocephala*	not decurrent	narrower or muchnarrower than culmsheath apex	oblong	at apex	glabrous

**Table 3:** Vegetative morphological characters and their states used in cluster analysis.

Number	Characters	Character statement	Note
01	Rhizomes length (cm)	Quantity	1–3 year-old rhizomes of 3 individuals
02	Culm height (m)	Quantity	
03	Culm top erect	Binary	
04	Pith lamella	Binary	
05	Node prominent	Binary	
06	Culm sheath yellow	Binary	
07	Culm sheath long trigon	Binary	
08	Culm sheaths deciduous	Binary	
09	Seta number per vision on culm sheaths	Quantity	
10	Hair length on culm sheaths	Quantity	
11	Culm sheath apex prominent	Binary	
12* †	Culm sheath blade base narrower than culm sheath apex	Binary	
13* †	Culm sheath blade base deccurent	Binary	
14	Culm sheath blade erect	Binary	
15	Number of branch per node	Quantity	
16	Number of leaf per final branch	Quantity	2 final branches per node
17	Ciliate at leaf sheath margin	Binary	Outer margin
18	Ridged on leaf sheath	Binary	New leaf sheath
19* †	Oral setae at apex of leaf auricle	Binary	Outer margin
20	Number of oral setae on leaf sheath	Quantity	Outer margin
21* †	Shape of leaf auricle	Multiple	Outer margin
22	Length of leaf sheath auricles	Quantity	Outer margin
23	Ciliate on ligule	Binary	
24	Out ligule notable	Binary	
25	Number of hair per vision on petiole	Quantity	
26	Ratio of the blade length and width	Quantity	
27	Leaf base cuneiform	Binary	
28	Leaf apex short acuminate	Binary	
29†	Number of hair per vision on blade back	Quantity	

*: Key characters;

† : Major characters; Quantity: quantity characteristics; Binary: binary characters; Multiple: multiple characters.

Three annual and three perennial culms were randomly selected in each individual and the vegetative morphological characters were measured in the field. Each of the characters was measured from the basal five nodes of three culms in a clump. For the characters from the sheathes, the measurement was carried out on the annual culms (bamboo shoots), and for the characters from the culms, branches and leaves, the measurement was carried out on the culms more than three-year-old (perennial culms), the characters of branch and leaf measure from the basal five nodes which have branches. The number of the hires on the culm sheathes and leaf blades was calculated from three 10 * 4.5 visions (about 1/20.25 square millimetres) with the stereomicroscope in three different areas for each of twenty sheathes or leaves on perennial culms and for all of sheathes and leaves on seedlings. Spring and summer leaves for perennial culms were measured separately.

The cluster analysis of morphological data from ten populations of the ingroup and two populations of the outgroup was carried out with UPGMA method using NTSYSpc 2.1 software package (Exeter Software, NY, USA).

### DNA isolation, amplification, sequencing and analyses

Total DNA was extracted from silica gel-dried leaves using a modified CTAB procedure [21]. The concentration of genomic DNA was determined by electrophoresis on 1.0% agarose gels. The DNA samples were diluted by ddH_2_O and stored at −20°C for use. The nrDNA ITS region was amplified by primers ‘ITS4’ and ‘ITS5’ from White *et al.* [22] (1990) and Guo *et al.* [23]. PCR amplifications of both chloroplast DNA fragments and ITS region were conducted in a 25-µL volume containing 1×PCR Buffer, 2 mM Mg^2+^, 250 µM each of dNTPs, 0.8 mM of each primer and 50–100 ng genomic DNA. The reactions were performed in an ABI Veriti™ 96-well Thermal cycler (Applied Biosystems, CA, USA), with the following program: initial denaturation at 95°C for 4 min; 35 cycles of 95°C for 45 s, 55°C for 1 min, 72°C for 1 min; and last synthesis at 72°C for 7 min. PCR products were detected on a 1% agarose gel, stained with ethidium bromide and visualized under UV light. The products were subsequently purified and sequenced by Beijing Dingguo Changsheng Biotechnology Co. Ltd. (Beijing, China).

Chromas Lite v2.01 (http://www.technelysium.com.au/chromas_lite.htm) was used to check the quality of the complete sequences. Sequence divergences between taxa and base frequencies (G+C content) were determined using MEGA v5.05 [24]. Neighbor-joining (NJ) tree was also calculated using MEGA v5.05 [24]. Phylogenetic analyses were performed using PAUP v4.0b10 [26].

## Results

### Analysis of key characters in ten populations

#### Variation on culm sheaths

**Figure 2:**
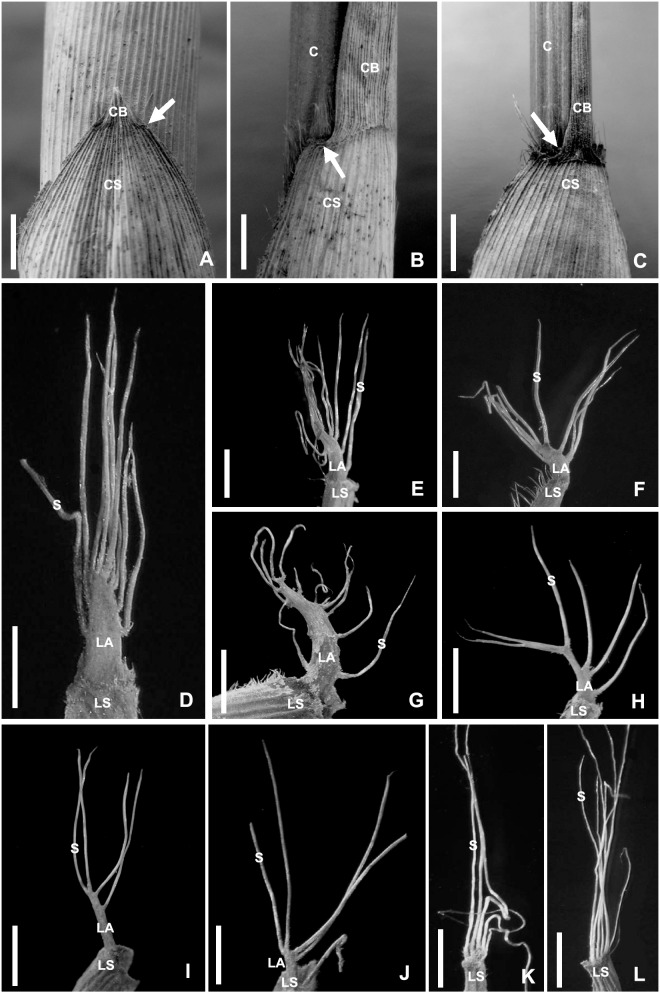
A–C. Close up of upper part of sheathes and lower part of sheath blades in different of a same annual culm in Pop 4. Bar = 5 mm. A. In lower part, showing blade base decurrent (arrow) and as wide as sheath apex. B. In middle part, showing blade base not decurrent (arrow) and narrower than sheath apex. C. In upper part, showing blade base not decurrent (arrow) and much narrower than sheath apex. D**–**L. Close up of leaf auricles and oral setae, showing the shape of leaf auricles and position of oral serae. Bar = 1 mm. D**–**F. From Pop 1. D**–**E. From an annual culm. D. Long triangular auricle with serae at upper margins and apex. E. Curving long triangular auricle with setae at margins and apex. F. Short sickle-like with setae mostly at one margin and apex from a perennial culm. G**–**H. From Pop 3. G. Sickle-like auricle with setae mostly at one margin and apex from an annual culm. H. Very narrow sickle-like auricle with setae mostly at one margin and apex from a perennial clam. I**–**J. Culms from Pop 8. I. Banding auricle with setae at upper margins and apex from an annual culm. J. Short banding auricle with setae at upper margins and apex from a perennial culm. K**–**L. From Pop 10. K. very small auricle with setae at whole margin from an annual culm. L. Almost without auricle but with setae on upper margin of sheath from a perennial culm. C: culm. CB: culm sheath blade. CS: culm sheath. LA: leaf auricle. LS: leaf sheath. S: oral seta.

**Figure 3:**
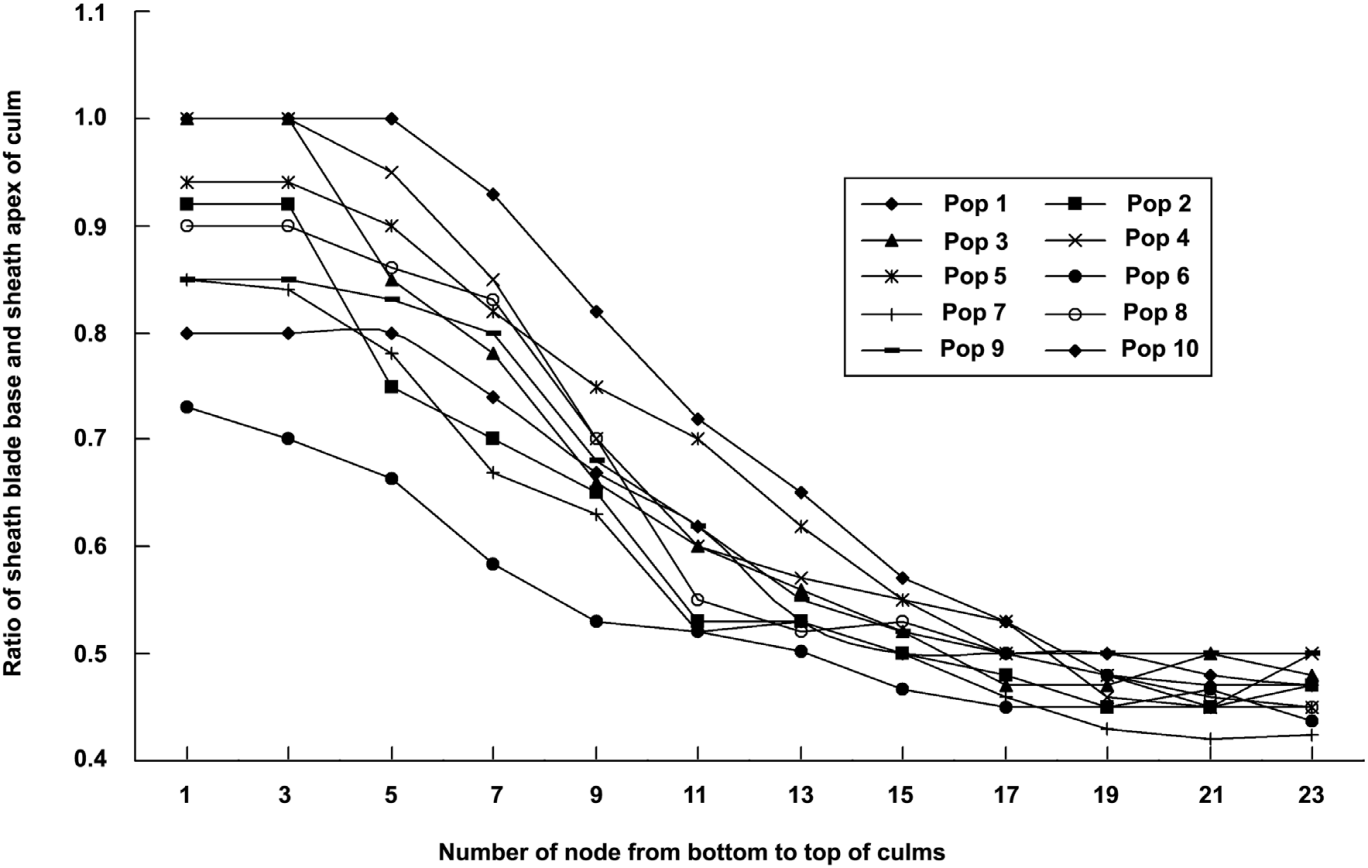
The variation of base width of culm sheath blade on a single bamboo shoot in different populations.

The two characters from the culm sheath vary in different nodes on a same bamboo shoot from the bottom to the apex, the blades change from short, triangular to long and ribbon or even leaf like, their bases change from as wide as the sheath apex, decurrent to narrower than sheath apex and not decurrent. Here we selected a culm from Pop 4 to illustrate the changes of the shape and base of sheath blades. The sheath blade is very small, triangular, the base is not decurrent and usually as wide as the sheath apex or nearly so in the third node (Fig. 2-A), the sheath blade is ribbon like, its base is also decurrent but is narrower than the sheath apex in the ninth node (Fig. 2-B), the sheath blade is longer, ribbon (or even leaf like in other culms of the same individual or in other populations), the blade base is not decurrent and much narrower than the sheath apex in the twenty first node (Fig. 2-C). Here we used the ratio of the width of the blade base and the width of the sheath apex to exhibit the change of the blade bases in different populations. The ratio of blade base and sheath apex is from 0.7 to 1.0 in the third node, from 0.53 to 0.82 in the ninth node, from 0.42 to 0.5 in the twenty first node. The ratio changes slowly from the first to the fifth nodes, while decreases gradually from the fifth to the seventeenth nodes, and then changes slowly again from the seventeenth to the twenty third nodes in any of ten populations (Fig. 3).

#### Variation on leaf auricles

Based on the observation on the types of *F*. *decurvata* (Lu Jionglin 78120, PE) and *F*. *dracocephala* (Yi Tong-pei 75540, Herb. Forestry School of Sichuan Province), the leaf auricles are short sickle-like, banding, short banding, but not elliptical, long elliptical or nearly circular, therefore we did not use “elliptical”, “long elliptical” and “nearly circular” to describe the shape of the auricles.

The shape of the auricles and the position of the oral setae are various in different populations, different individuals in the same population and even on a same culm, long triangular with setae at upper margins and apex (Fig. 2-D), curving long triangular with setae at margins and apex (Fig. 2-E), short sickle-like with setae mostly at one margin and apex (Fig. 2-F), sickle-like with setae mostly at one margin and apex (Fig. 2-G), very narrow sickle-like with setae mostly at one margin and apex (Fig. 2-H), banding with setae at upper margins and apex (Fig. 2-I), short banding with setae at upper margins and apex (Fig. 2-J), very small with setae at whole margin (Fig. 2-K), and almost without auricle but with setae on upper margin of sheath (Fig. 2-L). The field observations showed that the auricles and oral setae exist on all newly formed leaves while fall off on perennial leaves in some cases.

#### Variation on pubescence on blade back

It seems that the pubescence on blade back is an important difference between two species among the expanded characters, so more attention was paid on this character in the present study.

**Figure 4:**
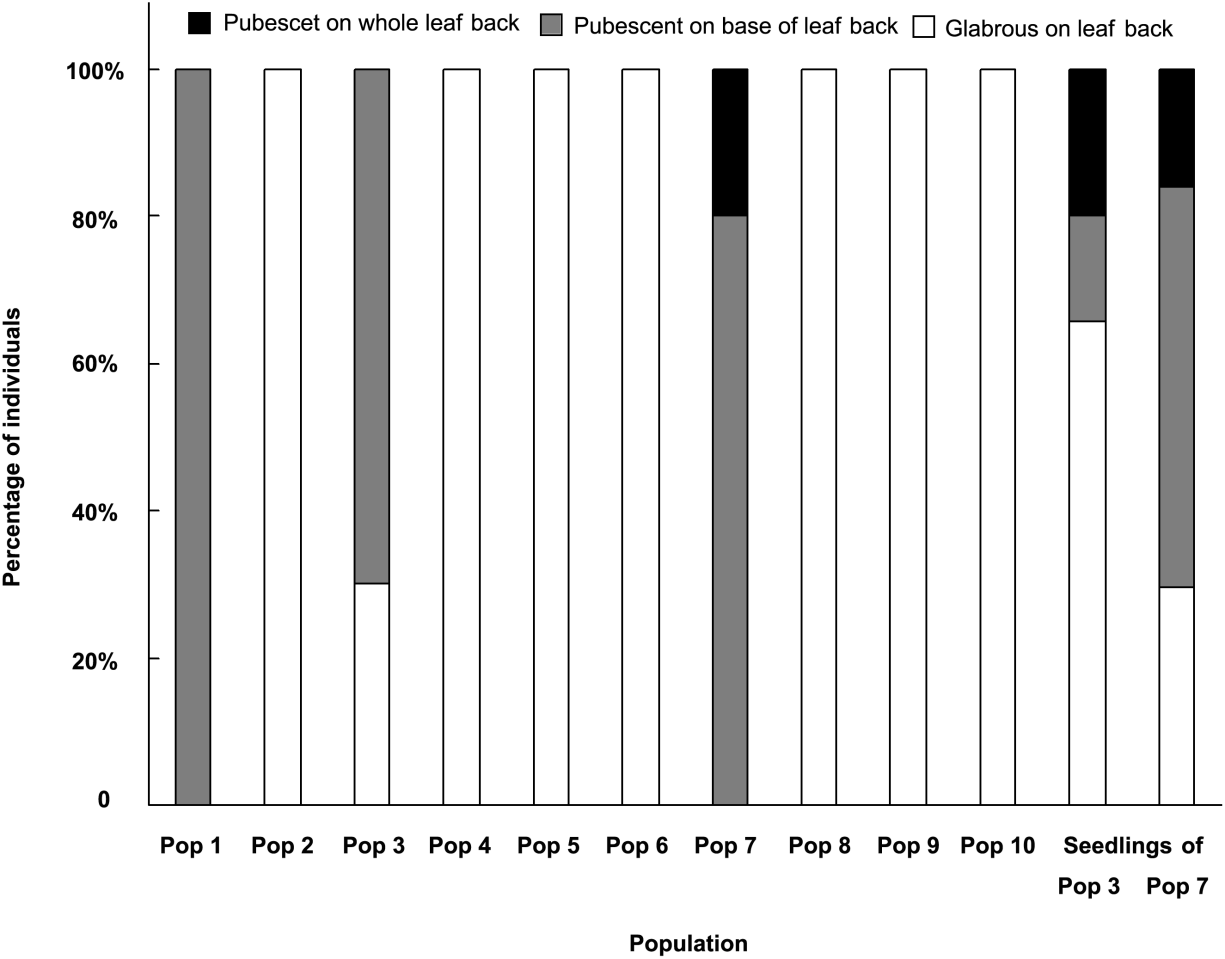
The percentage of individuals with different pubescent situation on leaf back in different populations.

The pubescence on blade back is various: glabrous on blade back, pubescent at the base of the blade back and pubescent on the whole blade back. In most of the populations, all individuals have the same status of the leaf pubescence in the same population, but in other populations different individuals have different status of the leaf pubescence in the same population (Fig. 4). The leaf pubescence is also various on the leaves from different seasons. For example, there are an average of 90.3 hires in a vision on spring leaves and an average of 125.5 hires on summer ones in Pop 3.

### Cluster analysis of vegetative characters

**Figure 5:**
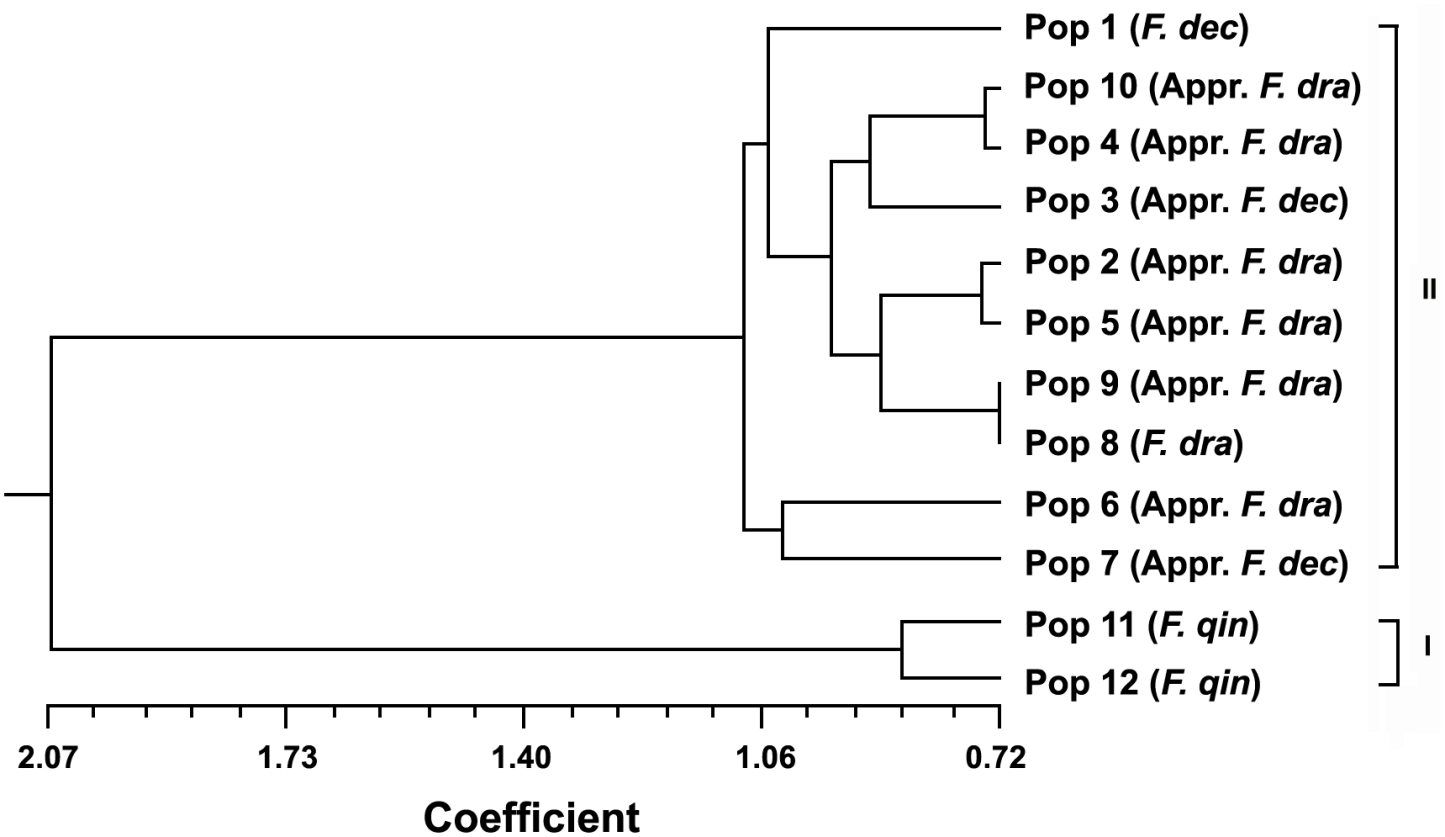
The UPGMA cluster result of the 29 vegetative characters. (Appr. = approximate; *F*. *dec* = *Fargesia decurvata*; *F*. *dra* = *F*. *dracocephala*; *F*. *qin* = *F*. *qinlingensis*).

The UPGMA cluster result (Fig. 5) of five major characters plus 24 expanded ones showed that twelve populations can be divided into two groups. Group I included two populations of *F. qinlingensis* as the outgroup, while the remaining populations of *F. decurvata* and *F. dracocephala* formed Group II. In addition, Group II can be divided into two subgroups. Pop 6 and Pop 7 constituted the first subgroup, and the second one comprised of the other eight populations. Among Group II, according to the key characters, Pop 1 was and Pop 3 and Pop 7 were approximate to *F*. *decurvata*, and the others were approximate to *F*. *dracocephala*. Therefore, the cluster analysis on the vegetative characters showed that the populations of these two species were nested one with another.

### Comparison of the floral organs

There is no difference either between any of two populations or between the flowers that we collected and the descriptions of *F. dracocephala* [7] on floral organs.

### The relationship based on the ITS sequence

Alignment of all the ITS region sequences of *F. decurvata* and *F. dracocephala* resulted in a matrix of 618 positions, including ITS1, ITS2 and 5.8s. Characteristics of these sequences, including length, G+C content, number of indels and variable bases, are summarized in Table 4. The length of ITS1 and ITS2 is 213 bp, and 216 bp, respectively. ITS1, with a maximum of 2.0% divergence across all taxa (1.9% in *F*. *decurvata* and *F*. *dracocephala*), is very slightly shorter and less variable in length than ITS2, which has a maximum of 2.0% divergence across all taxa (2.0% in *F*. *decurvata* and *F*. *dracocephala*). The 5.8S is 163 bp long in all taxa. As expected, the 5.8S gene showed little variation, with 1.2% divergence across all taxa. The overall of ITS divergence was approximately 1.86% within *F*. *decurvata* and *F*. *dracocephala*.

**Table 4:** Sequence characteristics of the internal transcribed spacers ITS1 and ITS2, and of the 5.8S subunit of nuclear rDNA of *F. decurvata*, *F. dracocephala* and *F. qinlingensis.*

Sequence characteristics	ITS1	5.8S	ITS2
Length range in all taxa (bp)	213	163	216–217
Length range in *F. decurvata and F. dracocephala* (bp)	213	163	216
Length range of outgroup species (bp)	213	163	217
Aligned length (bp)	213	163	217
G + C content range (mean) in *F. decurvata and F. dracocephala* (%)	71.3–81.7(71.5)	58.8	74.2–74.5(74.4)
G + C content range (mean) in all taxa (%)	71.3–71.7(71.5)	58.8	74.2–74.5(74.4)
Sequence divergence in *F. decurvata and F. dracocephala* (%)	0–1.9	0–1.2	0–2
Sequence divergence in all taxa (%)	0–2	0–1.2	0–2
Size of indels in *F. decurvata and F. dracocephala* (bp)	0	0	0
Size of indels in all taxa (bp)	0	0	1
Number (and %) of constant sites	208(97.6)	161(98.7)	210(96.8)
Number (and %) of variable sites	5(2.4)	2(1.2)	7(3.2)
Number (and %) of parsimony informative sites	4(1.9)	2(1.2)	5(2.3)

**Figure 6:**
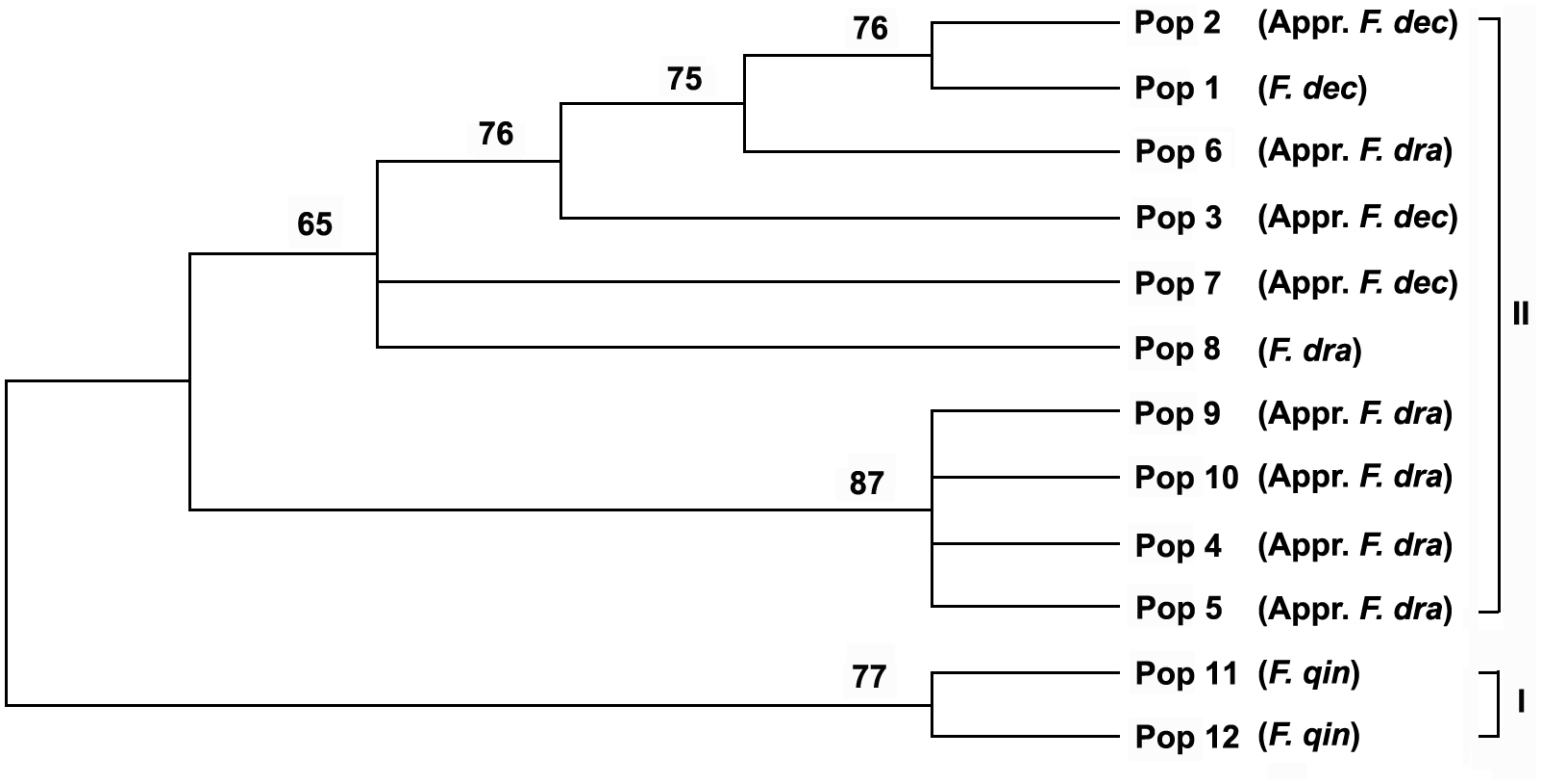
Strict consensus tree of the 43 most parsimonious trees of 324 steps derived from equally weighted maximum parsimony analysis of ITS sequences of ten populations of *F. decurvata* and *F*. *dracocephala* and two outgroup populations (CI = 0.823, RI = 0.808). Number above branches is bootstrap values; values <50% are not indicated; the abbreviation is same as that in Fig. 5.

**Figure 7:**
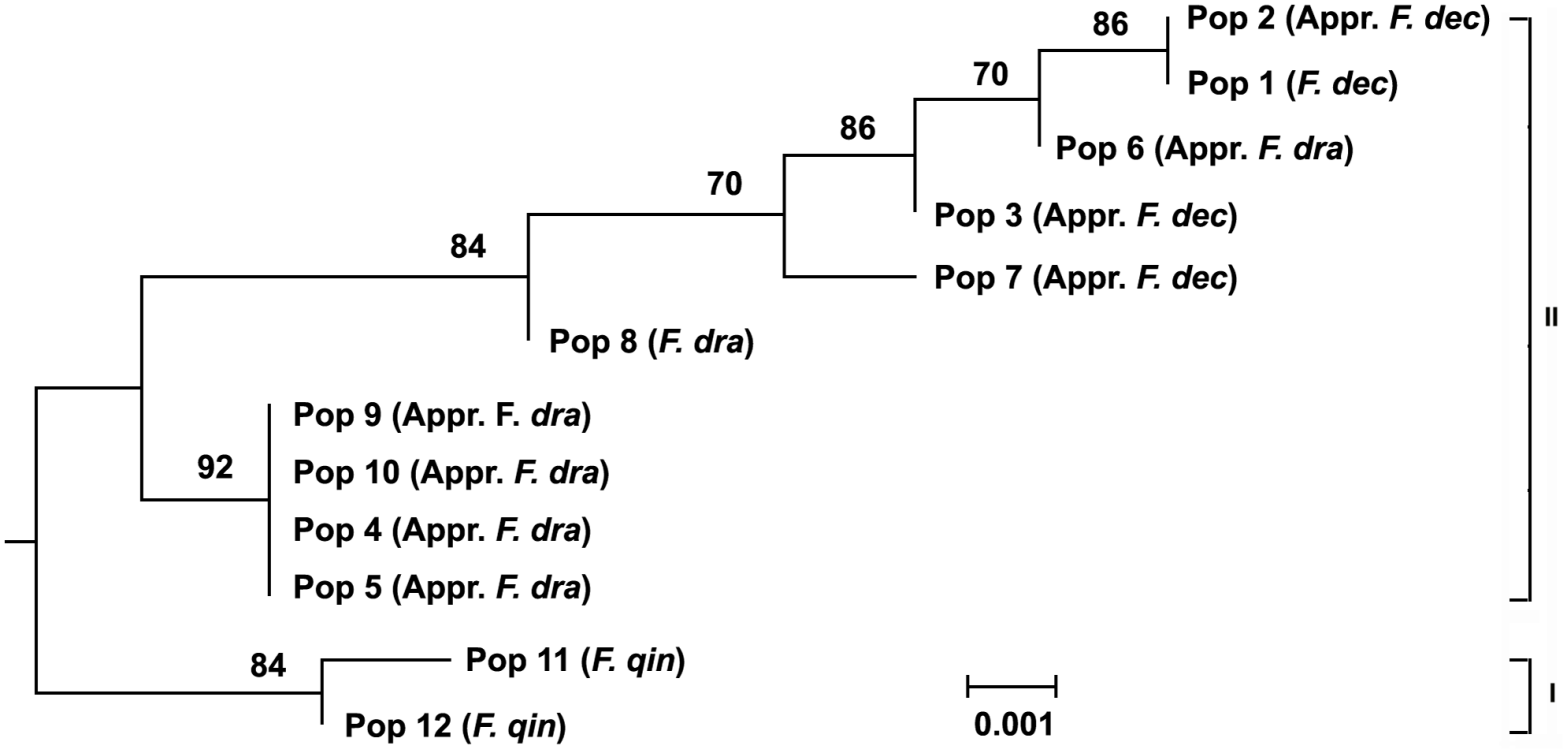
Neighbor-joining tree of the ten populations of *F. decurvata* and *F*. *dracocephala* and two outgroup populations inferred from ITS sequence. Number above branches is bootstrap values; values <50% are not indicated; the abbreviation is same as that in Fig. 5.

Based on the ITS sequence parsimony informative sites, both the MP tree and the NJ tree (Figs. 6 and 7) resulted in two major clades (I and II), with more than 75% bootstrap support. Two populations of *F. qinlingensis* formed clade I and the populations of *F*. *decurvata* and *F*. *dracocephala* constituted clade II. Clade II was mainly divided into two subcaldes. Pop 4, Pop 5, Pop 9 and Pop 10 of approximate *F*. *dracocephala* formed the first subclade. The second subclade is constitutive of the others populations (including *F*. *decurvata* and *F*. *dracocephala*); therefore, the populations of *F*. *dracocephala* were not included in the same subclades and that of *F. decurvata* were nested within that of *F. dracocephala*. Hence, according to the phylogenetic trees based on ITS sequence data, the present study did not support the ten populations to be separated as two species.

## Discussion

### On taxonomy of *Fargesia decurvata* and *F. dracocephala*

According to the previous description [7]–[12], the differences between the two species are: the culm sheath apex is promontory, the blade base of culm sheath is decurrent and is as wide as the apex of culm sheath, the leaf auricle is nearly circular with setae at the margin, pubescent on lower surface of leaf blade in *F. decurvata*; while the blade base of culm sheath is not decurrent and narrower or much narrower than the apex of culm sheath, the leaf auricles is elliptic with setae at the apex, glabrous on lower surface of leaf blade in *F. dracocephala*. Among the ingroup populations in the present studies, Pop 1 was collected from the type locality and the vegetative characters match completely with those of the type specimen of *F*. *decurvata* we observed in PE (Lu Jionglin 78120), Pop 8 was collected from the type locality and the vegetative characters match with those of *F*. *dracocephala* except the shape of the leaf auricle and pubescence. Based on the comparative analyses on 10 populations, each of the above mentioned characters is continuous or has intermediate status between two species. The continuous variation of characters occurs not only among populations, but also among individuals within a population, among different culms of a same individual and even in different parts of a same culm. The result from the cluster analysis of multiple vegetative characters, which was successful in the studies of allied species [2], [3], [27]–[29], showed that ten populations were not divided into two species as well. Therefore, it is impossible to distinguish these two species by using vegetative characters if the sampling goes beyond the type localities and if the key characters could not distinguish two species well, not multiple ones.

The characters of the flowers and fruits play an important role in the definition of species in angiosperms as well as in bamboos although it is difficult to obtain the flowers and fruits in bamboos [30]–[33]. In the present studies, the flowers were collected in Pop 3, Pop 7 and Pop 9, among them Pop 3 matches *F*. *decurvata* well in vegetative organs. The floral characters are the same in three populations and no difference with the floral description of *F*. *dracocephala* [7], [8], [12]. Therefore, it is obvious that there is no difference on the floral organs between these two species.

ITS sequence has been used widely in the studies of inter- and intra-specific relationships [22]–[24], [34], [35]. In the present study, the relationship of ten populations from *F*. *decurvata* and *F*. *dracocephala* and two populations from *F*. *qinlingensis* as the outgroup was established by using ITS sequence, with the credible bootstraps support (Figs. 5 and 6). The length and levels of variability of the ITS sequence accorded with the previous studies on bamboos [23], [36], [37]. The tree from the ITS sequence showed that twelve populations were clustered into two clades. The first clade included two populations of *F*. *qinlingensis*, and the second comprised ten populations of *F*. *decurvata* and *F*. *dracocephala*. This result indicated that the difference among ten populations from *F*. *decurvata* and *F*. *dracocephala* was less than that between ten populations and the outgroup. In the second clade, ten populations from *F*. *decurvata* and *F*. *dracocephala* were nested one with another. Although the ten populations were included into two clades, but none of them matched any species, and the two populations (Pop 1 and Pop 8) from the type localities were included in the same clade. Therefore, the analysis on the ITS sequence did not support the separation of *F*. *decurvata* and *F*. *dracocephala*.

### Taxonomic treatment

Based on the analyses of the morphological characters and ITS sequence, we considered that the description of *F*. *dracocephala* might be the result of discontinuous sampling of *F*. *decurvata* and *F*. *dracocephala* should be one species, and the name *F*. *dracocephala* should be treated as the synonym of *F*. *decurvata*.

*Fargesia decurvata* J. L. Lu in Journ. Henan Agr. Coll. 1981 (1): 74. Pl. 6. 1981; Flora Reipublicae Popularis Sinicae 9 (1): 471. Pl. 132: f. 16, 17. 1996; Flora of China 22: 93. 2006–*F. aurita* Yi in Journ. Bamb. Res. 4 (4): 22. pl. 6. 1985 et in ibid. 7 (7): 15. 1988–*F. dracocephala* Yi in Bull. Bot. Res. 5 (4): 127. f. 4. 1985 et in Journ. Bamb. Res. 7 (2): 15. 1988 et in ibid. 9 (1): 32. f. 3. 1990. adjust. flor. Descry; Flora Reipublicae Popularis Sinicae 9 (1): 469. Pl. 132: f. 1–13. 1996; Flora of China 22: 93.

### On some characters used in key of species in *Fargesia*

The characters used to distinguish *F*. *decurvata* and *F*. *dracocephala*, such as with or without and the shape of the leaf auricle, the pubescence on leaf blade back, and the position of the oral serae, were frequently used in the key of species in *Fargesia*, especially in Ser. *Yunnanenses*, Ser. *Angustissimae*, and Ser. *Fargesia* [7], [8]. Based on the present studies, these characters are unstable because they vary not only between populations, but also between individuals within a population, and even between culms of an individual or different position of a same culm. Some of the characters may vary in different developmental stages or different age. The rationality of the species based on such characters might be doubtable. Although there have been no evidence to show that many of the characters used in the key of species are continuous between or among species, but fact is that many tiny morphology characters were used in distinguishing two or even groups of species and one cannot find any biological or ecological meaning.

### On sampling in definition of bamboo species

It is well know that a species is composed by population(s), and there has more or less difference(s) between populations because of the exiting of the phenotypic plasticity and genetic differentiation [3], [27], [29], [38]. The phenotypic plasticity and genetic differentiation within a species might lead to the continuous variations of morphological characters. The phenotypic plasticity and genetic differentiation might be ignored and the continuous variation of the morphological characters might be isolated artificially within a species in case of discontinuous sampling. This might lead to the result that many microspecies were described and published. The result from the present studies showed that *F*. *dracocephala* is one of the variations of *F*. *decurvata* and the publication of *F*. *dracocephala* is the result of the discontinuous sampling of *F*. *decurvata*. The cases of discontinuous sampling which made the morphological characters were isolated artificially was also found in either bamboo or other taxa [2], [3], [39], [40], for example, *Ph. sapida* Yi (Bambusoideae, Poaceae) was proved to be one of the ecotypes *Ph. flexuosa* A. et C. R. cased by the environment changes after comparing a large number of specimens [41], ten species in *Cornus schindleri* complex (Cornaceae) collected from Kongding, Sichuan Province were proved to be two subspecies of *C*. *schindleri* [29]. In fact, if four or more morphological characters, such as four key characters between *F*. *decurvata* and *F*. *dracocephala*, or some tiny morphological characters, such as pubescence situation on the leaves, which is the only difference between some species or even two groups of species in *Fargesia* [7], have to be used in the distinguish of two or more allied bamboo species, then it is reasonable to doubt that the so called allied species might be microspecies causing by the discontinuous sampling of the same species.

In the definition of species, after all, the specimen is numbered. The species itself is a collection of groups with a typical character, species defining is the delimitation of the characters of the group back together [42]–[44], and the specimen is only a reference point on the naming. Often in the case of insufficient number of specimens, the continuous variation characters will be presented in the form of intermittence, causing difficulties and even errors in classification, and when the observed populations are too small or the observation is intermittent, it will also definitely lead to fragmentation of the continuously changing characters. So in the species defining, as Nooteboom [4] stated “It is always necessary to study as many collections as possible from numerous herbaria, not only from a restricted area but from the whole area where a taxon may occur”.

## Supporting Information

Table S1 **The vegetative character matrix.**
(DOC)
