# Supplementary material for: Merging Fargesia dracocephala into Fargesia decurvata (Bambusoideae, Poaceae): Implications from Morphological and ITS Sequence Analyses
Source: PLoS One. 2014 Jul 2;9(7):e101362. doi: 10.1371/journal.pone.0101362 (PMC4079244; doi:10.1371/journal.pone.0101362)
Supplement: Table S1 — The vegetative character matrix. (DOC) [file pone.0101362.s001.doc]

Table S1. The vegetative character matrix

| Character No. | Pop 1 | Pop 2 | Pop 3 | Pop 4 | Pop 5 | Pop 6 | Pop 7 | Pop 8 | Pop 9 | Pop 10 | Pop 11 | Pop 12 |
| --- | --- | --- | --- | --- | --- | --- | --- | --- | --- | --- | --- | --- |
| 01 | 20 | 22 | 24 | 18 | 20 | 26 | 22 | 20 | 20 | 15 | 14 | 18 |
| 02 | 2.5 | 2.3 | 2.5 | 1.8 | 2.8 | 2.8 | 2.1 | 2.2 | 1.8 | 1.5 | 2.5 | 2.7 |
| 03 | 1 | 1 | 1 | 1 | 1 | 1 | 1 | 1 | 1 | 1 | 0 | 0 |
| 04 | 1 | 1 | 1 | 1 | 1 | 1 | 1 | 1 | 1 | 1 | 0 | 0 |
| 05 | 1 | 1 | 1 | 1 | 1 | 1 | 1 | 1 | 1 | 1 | 0 | 0 |
| 06 | 1 | 1 | 1 | 1 | 1 | 1 | 1 | 1 | 1 | 1 | 0 | 0 |
| 07 | 1 | 1 | 1 | 1 | 1 | 1 | 1 | 1 | 1 | 1 | 0 | 0 |
| 08 | 0 | 0 | 0 | 0 | 0 | 0 | 0 | 0 | 0 | 0 | 1 | 0 |
| 09 | 40 | 0 | 70 | 60 | 30 | 140 | 20 | 0 | 20 | 20 | 40 | 80 |
| 10 | 1.4 | 0 | 1.6 | 1.6 | 1.3 | 1.8 | 1.4 | 0 | 1.5 | 1.2 | 1.2 | 1.5 |
| 11 | 0 | 0 | 0 | 0 | 0 | 0 | 0 | 0 | 0 | 0 | 1 | 1 |
| 12 | 0 | 0 | 0 | 0 | 0 | 0 | 0 | 0 | 0 | 0 | 1 | 1 |
| 13 | 0 | 0 | 0 | 0 | 0 | 0 | 0 | 1 | 1 | 0 | 1 | 1 |
| 14 | 0 | 0 | 0 | 0 | 0 | 0 | 0 | 0 | 0 | 0 | 1 | 1 |
| 15 | 13 | 8 | 9 | 9 | 10 | 9 | 8 | 9 | 10 | 9 | 5 | 5 |
| 16 | 3 | 3 | 3 | 3 | 3 | 3 | 3 | 3 | 3 | 3 | 2 | 2 |
| 17 | 2 | 1 | 1 | 1 | 1 | 2 | 2 | 1 | 2 | 1 | 2 | 2 |
| 18 | 0 | 0 | 1 | 0 | 0 | 0 | 1 | 1 | 0 | 0 | 1 | 1 |
| 19 | 1 | 1 | 1 | 1 | 1 | 1 | 1 | 1 | 1 | 1 | 0 | 0 |
| 20 | 11 | 6 | 8 | 7 | 8 | 5 | 7 | 8 | 6 | 9 | 9 | 6 |
| 21 | 5.3 | 3.1 | 3.9 | 4.6 | 3.6 | 3.0 | 3.5 | 5.4 | 4.0 | 4.3 | 3.3 | 2.1 |
| 22 | 1.21 | 1.03 | 1.08 | 1.63 | 0.74 | 0.72 | 1.02 | 1.22 | 1.10 | 1.17 | 0.50 | 0 |
| 23 | 0 | 0 | 1 | 1 | 0 | 0 | 0 | 0 | 0 | 1 | 0 | 0 |
| 24 | 0 | 1 | 1 | 0 | 0 | 0 | 0 | 1 | 1 | 0 | 0 | 0 |
| 25 | 48 | 0 | 24 | 0 | 0 | 0 | 50 | 0 | 0 | 0 | 0 | 0 |
| 26 | 12.5 | 11.2 | 11.2 | 10.3 | 10.4 | 12.5 | 11.8 | 12.2 | 11.7 | 13.7 | 9.3 | 9.2 |
| 27 | 1 | 1 | 1 | 1 | 1 | 1 | 1 | 1 | 1 | 1 | 0 | 0 |
| 28 | 0 | 1 | 0 | 1 | 1 | 0 | 1 | 0 | 0 | 0 | 1 | 1 |
| 29 | 0 | 0 | 10 | 0 | 0 | 0 | 30 | 0 | 0 | 0 | 0 | 0 |
